# Lobular carcinoma in situ (LCIS) of the breast: is long-term outcome similar to ductal carcinoma in situ (DCIS)? Analysis of 200 cases

**DOI:** 10.1186/s13014-015-0379-7

**Published:** 2015-05-06

**Authors:** Bruno Cutuli, Brigitte De Lafontan, Youlia Kirova, Hugues Auvray, Agnes Tallet, Sandrine Avigdor, Claire Brunaud, Catherine Delva

**Affiliations:** Institut du Cancer Courlancy, Reims, France; Institut Claudius Regaud, Toulouse, France; Institut Curie, Paris, France; Centre Henri Becquerel, Rouen, France; Institut Paoli-Calmettes, Marseille, France; Centre Hospitalier Régional, Orleans, France; Institut de Cancerologie de Lorraine, Vandoeuvre-les-Nancy, France; Sylia-Stat, Bourg-la-Reine, France

**Keywords:** Lobular carcinoma in situ, Breast cancer, Treatment, Local recurrence, Radiotherapy, Breast-conserving surgery

## Abstract

**Background:**

Lobular carcinomas in situ (LCIS) represent 1-2% of all breast cancers. Both significance and treatment remain widely debated, as well as the possible similarities with DCIS.

**Materials and methods:**

Two hundred patients with pure LCIS were retrospectively analyzed in seven centres from 1990 to 2008. Median age was 52 years; 176 patients underwent breast-conserving surgery (BCS) and 24 mastectomy. Seventeen patients received whole breast irradiation (WBRT) after BCS and 20 hormonal treatment (15 by tamoxifen).

**Results:**

With a 144-month median follow-up (FU), there were no local recurrences (LR) among 24 patients treated by mastectomy. With the same FU, 3 late LR out of 17 (17%) occurred in patients treated by BCS and WBRT (with no LR at 10 years). Among 159 patients treated by BCS alone, 20 developed LR (13%), but with only a 72-month FU (17.5% at 10 years). No specific LR risk factors were identified. Three patients developed metastases, two after invasive LR; 22 patients (11%) developed contralateral BC (59% invasive) and another five had second cancer.

**Conclusions:**

LCIS is not always an indolent disease. The long-term outcome is quite similar to most ductal carcinomas in situ (DCIS). The main problems are the accuracy of pathological definition and a clear identification of more aggressive subtypes, in order to avoid further invasive LR. BCS + WBRT should be discussed in some selected cases, and the long-term results seem comparable to DCIS.

## Background

Firstly described in 1941 by Muir, Foote and Stewart, the exact significance of lobular carcinoma in situ (LCIS), also called lobular neoplasia (LN), remains debated, ranging for many years from a simple marker of subsequent invasive carcinoma to, more recently, a real pre-cancerous lesion in many cases, especially invasive lobular carcinoma (ILC) [1,2].

The variability of pathological definitions according to time, with frequent combined analysis of LCIS/LN and atypical lobular hyperplasia (ALH) or new classification defined in 2002 and including three types of lobular intra-neoplasia (LIN), as well as rarity and frequent insufficient long-term follow-up in several series, all represent additional uncertainties regarding its optimal treatment [3].

Our study evaluated the long-term outcome in a cohort of 200 women with pure LCIS treated by three different options, i.e. breast-conserving surgery alone (BCS), BCS with whole breast irradiation (BCS + WBRT) or mastectomy (M). We compared our results according to the same treatments used in DCIS [4,5], particularly assessing the potentially life-threatening risk of invasive local recurrence (LR).

## Materials and methods

From January 1990 to December 2008, 200 patients with “pure” LCIS (without associated DCIS and/or previous or synchronous contralateral DCIS or invasive breast cancer) were retrospectively collected in seven centres, also sometimes including benign lesions, e.g. atypical (ductal and/or lobular) hyperplasia or sclerosing adenosis. Seven patients had bilateral synchronous LCIS. Median age was 52 years (range: 32–77).

79 out of 180 (44%) patients were post-menopausal and 45 of them (57%) underwent hormonal replacement therapy (HRT) for a 5-year median duration (76% by estrogen and progestins association).

In 60 out of 166 (30%) specified cases, a first and/or second family-history degree of breast cancer was found. Two patients had BRCA-1 and 2 mutation.

53 (44%) patients underwent previous surgery for a benign lesion, i.e. fibroadenoma (14), cyst (8), atypical hyperplasia (8) and sclerosing adenosis (2). There were 95 (46%) lesions in the right and 112 (54%) in the left breasts, respectively. Clinical symptoms were present in 44 (21%) cases. In 143 (72%) cases, LCIS was discovered by mammography and/or ultrasound (Table 1). In 15 cases, the lesion was discovered incidentally after surgery performed for benign disease or breast reduction.

**Table 1:** **Radiological features leading to biopsy (183 evaluable cases)**

	**n**	**%**
Microcalcifications (isolated)	105	57.4
Microcalcifications and other abnormalities	15	8.1
Round opacity	31	16.9
Stellar opacity	6	3.3
Abnormal density	12	6.6
Architectural distorsion	4	2.1
No abnormalities	10	5.6

## Treatments

### Surgery

168 patients underwent lumpectomy (7 with a complementary resection), 8 quadrantectomy and 24 (6 in two times) mastectomy (nine of whom had contralateral prophylactic mastectomy), due to multicentric disease or patient's preference.

### Radiotherapy

Seventeen patients underwent a classical 50 Gy/25 fractions WBRT, including 8 with a 10-Gy boost. There were no factors influencing the WBRT choice, i.e. age, LIN subtype, excision quality or multifocality.

### Hormonal therapy (HT)

Twenty patients received hormonal therapy (15 by Tamoxifen), 17 of them after BCS (10.7%), two after BCS + RT (11.8%) and only one after mastectomy. HT was significantly more prescribed in case of incomplete excision (16% versus 4.5%, p = 0.041) or LIN 3 or 2 versus 1 (43%, 15% and 4% respectively, p = 0.03).

### Pathology

All centres had pathologists deeply involved in the breast field, but central review could not be carried out. The subtype was only specified in 128 cases (classical in 124 and pleomorphic in 4).

According to LIN classification (applied since 2002), among our 94 evaluable cases, we found 25 LIN 1, 54 LIN 2 and 15 LIN 3. Moreover, among 172 evaluable cases, a single focus of LCIS was found in 101 patients (59%) and multiple foci in 71 (41%). The multiple foci rate was significantly higher (p = 0.001) in patients treated by mastectomy (77.3%) versus BCS (+/−WBRT) (36%). Among 143 evaluable cases, excision was considered complete in 112 (78%) cases, incomplete in 13 (9%) and doubtful in 18 (13%). Among 75 evaluable cases, the median lesion size was 12 mm.

Moreover, associated atypical epithelial hyperplasia (AEH), atypical lobular hyperplasia (ALH) and sclerosing adenosis were found in 68/187 (36%), 48/184 (26%) and 46/181 (25%) of the cases respectively.

## Results

### Local recurrences

**Figure 1:**
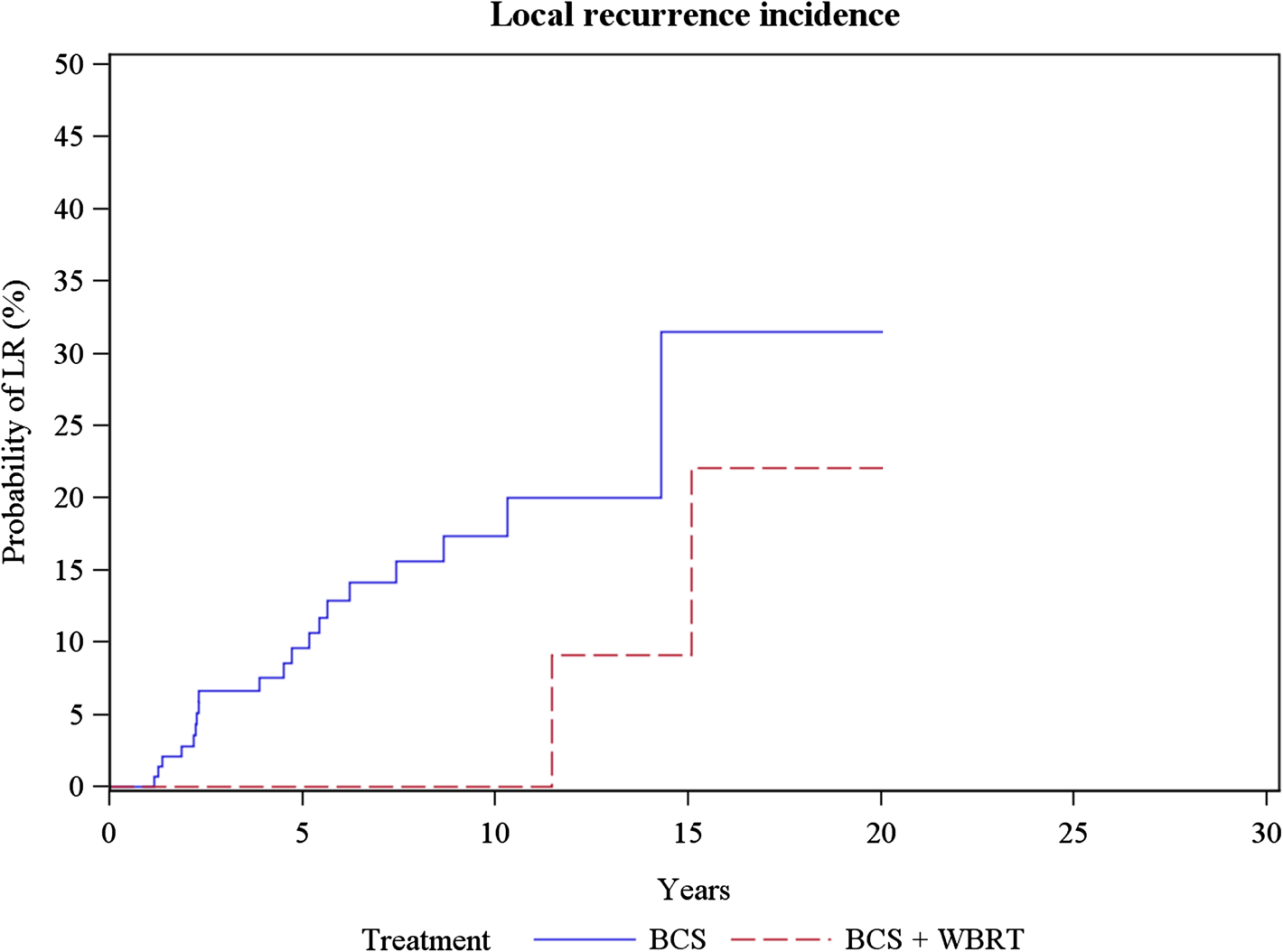
LCIS: Local recurrence incidence.

All patients had a bi-annual clinical FU in the first five years, then once a year. A mammography was also completed by ultrasound each year. With a 144-month median follow-up, there were no local recurrences (LR) among 24 patients who underwent mastectomy. With the same follow-up, three out of 17 (17%) patients treated by BCS + RT developed LR (all of them after 10 years), i.e. one DCIS, one infiltrating ductal carcinoma (IDC) SBR-1 pT_1_bN_0_ and one IDC SBR-3 pT_1_cN_1_. Among the BCS group with only a 72-month follow-up, 20/159 (13%) LR occurred (Figure 1). Sixteen (67%) were invasive, including 11 IDC, two tubular carcinomas and three ILC (one had previously recurred as LCIS). Among non-invasive LR, there were three DCIS and one mixed DCIS-LCIS. There were no significant clinical (mode of discovery, age) or pathological (number of foci, excision quality, AH presence) LR factors.

### Axillary recurrences

An associated axillary nodal involvement (ANI) was found in three cases (two ILC and one IDC). One patient treated by mastectomy developed axillary recurrence 20 years later certainly due to missed foci of invasive carcinoma in the mastectomy specimen. Thus, the overall axillary recurrence rate for the entire cohort was 2% (4/200).

### Metastases

Three patients developed metastases, two with previous invasive LR (one ILC and one IDC). Thus, the rate of metastasis after invasive LR was 11% (2/18).

### Contralateral breast cancer (CBC)

**Figure 2:**
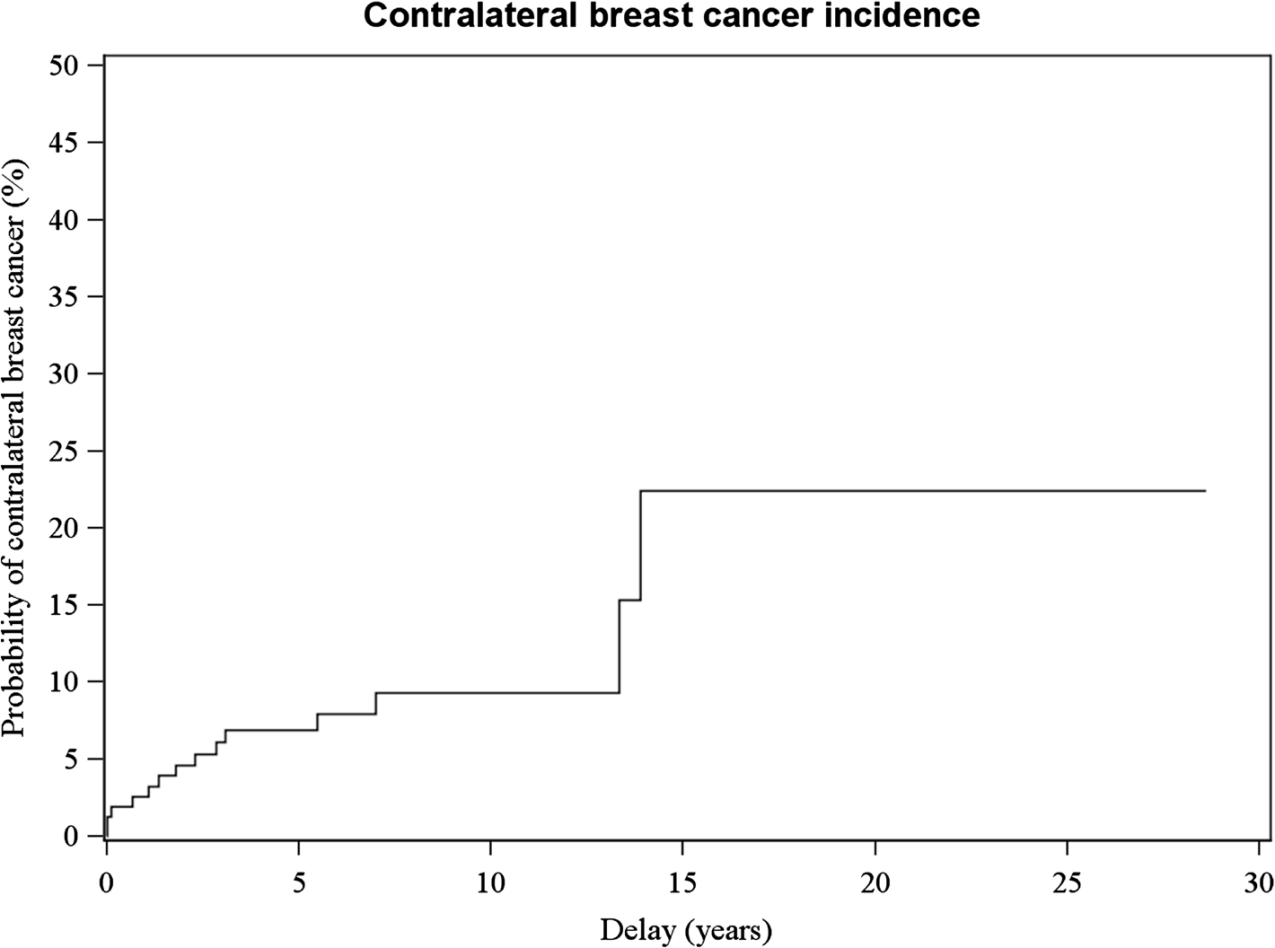
LCIS: Contralateral breast cancer incidence.

Twenty-two out of 191 patients developed CBC (11.5%) (Figure 2). The rate varies according to treatment and follow-up (Table 2). Overall, 13 out of 22 (59%) CBC were invasive.

**Table 2:** **Contralateral breast cancer (CBC) according to treatment**

	**n**	FU*	**%**	**In situ (n)**	**Invasive (n)**
BCS** (159)	15	72	9	4	10
BCS + WBRT*** (17)	3	144	17	3	-
Mastectomy (24)	5	144	21	2	3
Total (200)	22	87	11	9	13

*months.

**BCS: breast-conserving surgery.

***WBRT: whole breast irradiation.

### Second cancers

Five patients developed a second cancer (colon: 2, thyroid: 1, skin and salivary gland: one each).

### Long-term follow-up

Six patients were lost of follow-up and 189 are still alive. Four patients died, one by BC, one by intercurrent disease and two by colic cancer.

## Discussion

### Incidence and epidemiology

Due in large part to increased mammographic screening, DCIS incidence increased 7.2 fold in the USA from 1980 to 2001 and LCIS 2.6 fold [6,7]. Similar trends were observed in other Western countries [8]. The median age at diagnosis in most LCIS series varies from 45 to 50 years [1,8], i.e. 7–8 years younger than in the DCIS series [5]. Few data suggest a possible LCIS increase due to hormone replacement therapy (HRT) [6]. In our series, 57% of 79 menopausal women received HRT. Despite the possible discovery by clinical symptoms, very often due to benign associated lesions (cyst or fibroadenoma), our series confirms that about 65% of LCIS were discovered by clustered microcalcifications, such as reported by other studies [9,10]. From a pathological point of view, the distinction between LCIS and ALH is sometimes difficult and subjctive. Both lesions are often associated [11-13] and finally represent a temporal continuum in which the extent of lobular involvement varies [1]. Moreover, the loss of E-cadherin expression characterizes LCIS, facilitating a distinction from ductal lesions.

### Significance of LCIS and possible underestimation at core-needle biopsy (CNB)

For many years, LCIS (only representing 1-2% of all breast cancers) has just been considered a marker of risk for subsequent (in situ or invasive) cancer in both breasts [1]. The discovery by mammography or presence of another benign lesion leads to a complementary excision after CNB only in a variable part of cases, ranging from 40% to 70% in the literature and without clear selection criteria. Some authors proposed surveillance or complementary excision according to several and often different parametres, sometimes including «lobular neoplasia» encompassing both LCIS and ALH. In a large review by Hussain [2], including 31 studies, 789 out of 1229 (64%) patients diagnosed with LN on CNB had surgical excision: 280 were classified ALH, 241 LCIS, 22 pleomorphic LCIS (PLCIS) and 246 unspecified LN. In this review, the overall underdiagnosis rate.

(DCIS and/or invasive BC at subsequent excision) was 27% (211 out of 789). In a wide series from New York, among 201 out of 285 (71%) excisions performed for LN after CNB, 26 (13%) were upgraded; 7 had DCIS, 9 ILC and 10 IDC [14]. However, the risk of underestimated malignancy at subsequent excision (DCIS or infiltrating carcinoma) was only 8% (DCIS: 2.7% and infiltrating BC: 5.3%) among 478 lesions in a literature review reported by Murray, but with wide variations (from 3% to 35%) [15]. Finally, the initial underestimation risk after CNB showing LCIS or ALH is quite difficult to assess, without clear predictive criteria in the literature [16].

### Potential risk of LCIS invasiveness

Such as for DCIS, the potential LCIS risk is invasive BC development, keeping in mind that both lesions are theoretically initially fully curable. Compared to women in the general population, those with LCIS have a minimum 5–6 fold higher risk of IBC [9]. This risk increases in case of associated lesions (AH) [10] and especially when first-degree family history of BC was present [2], almost reaching the risk (8–10 fold) of women with BRCA 1–2 mutations. These data confirm the crucial importance of identifying the most «aggressive» LCIS subtypes leading to invasive BC.

### Comparisons between DCIS and LCIS treatments

DCIS now represent 15 to 20% of all breast cancers (63 000 cases in the USA in 2014) and are considered long-term precursors of invasive BC in 40-50% of the cases, even in low-grade lesions [5]. However, DCIS encompass several heterogeneous lesions due to many biological and molecular features leading to variable progressions to invasion. There are much more DCIS treatment data than for LCIS [13,17,18].

Mastectomy remains performed in 30-42% of DCIS according to the series [19,20], due to extension of the lesions, multicentricity and/or patients' preference. The local control rate was about 98% [4]. On the other hand, very few data are available on mastectomy rate in LCIS [7,13]. In our series, multicentricity seemed to be a factor leading to mastectomy.

In a recent American report using the Surveillance, Epidemiology and End Results (SEER) database, among 11 641 patients diagnosed with LCIS from 2000 to 2009, 16% underwent mastectomy, but with large differences based on geographic areas and age (from 12% to 24%) [7]. The local control rate, such as in our 24 patients, was almost 100%.

Due to more frequent discovevry of «small lesions», a conservative approach was gradually used in DCIS [17,18]. The standard treatment includes BCS and whole breast irradiation (WBRT) according to four large randomized trials (combined in a meta-analysis including over 4 500 cases) and retrospective studies showing that WBRT halfed the local recurrence rates (LR), both in situ and invasive [21-25]. Consequently, for these lesions, the 10-year LR rates are about 5-8% in the most recent series [26-28], reaching only 1% at 7 years in the very selected study on low-risk DCIS by RTOG [29]. However, in 20-25% of the cases, for various reasons, WBRT is not used in DCIS, leading, even in very selected cases (small lesions, wide margins, low grade), to a 15-20% 10-year LR rate, half of which invasive [30,31].

This is a crucial problem, because now several studies confirm the unfavourable impact of invasive LR in patients treated for DCIS with a 12-18% long-term of metastatic evolution [21,22,32]. For LCIS, there are no clear therapeutic guidelines, both after CNB and surgical excision [1,2,9]. The previously SEER-quoted study [7] showed that 10% of the patients had biopsy alone (precise criteria for the choice not specified) and 73% excision alone, whereas only 1% (116 patients) underwent complementary WBRT, without specific choice criteria or data on local control.

Table 3 shows the risk of subsequent invasive or in situ carcinoma after treatment of LCIS by excision alone in the main literature series [33-36]. This risk is quite similar in several DCIS series with the same treatment, i.e. San Francisco or Ontario series [30,31], as well as a very selected study by ECOG [37], with approximately 50% of invasive local recurrences.

**Table 3:** **Risk of subsequent invasive or in situ carcinoma after treatment of LCIS by excision alone**

**Author (year)**	**Ref**	**n**	FU**	**Invasive BC**		**DCIS**	
				**Total**	**%**	**Total**	**%**
Salvadori (1991)	[33]	78	58	5	6,4	-	-
Ottesen (1993)	[34]	69	61	8	11,6	4	5,8
Fisher E. (2004)	[35]	180	144	9	5	17	9,4
Chuba (2005)	[36]	3141*	120	NS	8,8	NS	NS

*SEER data.

**months.

In comparison with DCIS, the slope of the LR curve for LCIS seems quite less sharp and more spread out in time (see Figure 1), suggesting that the overall cancerization process was slower.The increase of local recurrence rates due to young age and incomplete excision (close and/or positive margins) is not clearly documented in LCIS compared to DCIS. However, few LCIS subtypes, e.g. pleomorphic LCIS, very extensive LCIS (with over ten involved acini) or LCIS with a great amount of necroses, seem to be clearly more aggressive lesions [3,9] and should be treated such as DCIS, as suggested by 2009 French Breast Carcinoma in Situ guidelines (www.e-cancer.fr).

Therefore, in these aggressive LCIS, WBRT addition should be discussed in the multidisciplinary team, in order to minimize the LR risk. In our previous report, we observed only one invasive LR out of 25 cases, with a 153-month follow-up [38]. In the present study, the LR is slightly higher (3/17), but the number of cases is lower and all LR occurred beyond 10 years.

These results are quite similar to DCIS treated by lumpectomy and WBRT in the series with a more than 120-month follow-up, both in retrospective studies and randomized trials [5,21-23]. However, it remains difficult to better identify the «aggressive» LCIS subtypes. A recent study by the Curie Insitute showed that KI67 should be used as a «discriminant» parameter to select more aggressive LCIS. Indeed, among 43 patients, the 5-year LR rates were 1/34 (3%) for low Ki 67 (≤10%) and 3/9 (33%) for high Ki 67 (>10%) (p = 0.0054) [39].

Another possible way of reducing the long-term risk of LR is the use of Tamoxifen. Indeed, the positivity rate of ER and/or PgR in LCIS is higher than in DCIS [9], and the results of the former NSABP P-1 prevention study showed that the most effective reduction of invasive BC by tamoxifen was observed among women with LCIS and ALH, suggesting a possible increased impact of anti-estrogens in the first steps of breast cancerization [40]. Similar results were observed in the NSABP P-2 prevention trial (STAR) using Raloxifen [41]. On the other hand, it should be remembered that the CBC rate in LCIS is quite higher than in DCIS, reaching 14% at 10 years in a large study from Connecticut tumor registry [42]. Consequently, the use of tamoxifen should be considered, especially in pre-menopausal women with a lower incidence of thrombo-embolic accidents and uterine cancers.

In short, LCIS is not always an indolent disease. Table 4 shows several features in comparison with DCIS. In fact, many data are quite similar with the almost same long-term LR rates, both after BCS alone and BCS + WBRT.The scarcity of this lesion urgently requires further larger studies, at least prospective long-term records, to clearly assess the LR and CBC rates according to radiological, clinical, histopathological and molecular features, in order to evaluate the potential benefit in selected cases of WBRT and tamoxifen [43].

**Table 4:** **Comparison between DCIS and LCIS (data adapted from references 1, 2, 5, 9, 13, 17, 36, 37)**

	**DCIS (%)**	**LCIS (%)**
Frequency	15-20	1-3
Calcifications at diagnosis	90	60-70
Excision rate after CNB	100	50-80
Hormone receptor positivity	70-80	90
Treatments:		
Mastectomy	30-40	10-15
BCS	20-25	80-85
BCS + WBRT	60-70	1-3
CBC	5-8	12-15
10-year LR (after BCS)	15-30	10-15
Invasive LR	50-60	30-40

BCS: breast conserving surgery, with wide variations in extent. CNB: core needle biopsy. CBC: contralateral breast cancer. LR: local recurrence (in situ and/or invasive). WBRT: whole breast irradiation.
